# Evidence of bacterioplankton community adaptation in response to long-term mariculture disturbance

**DOI:** 10.1038/srep15274

**Published:** 2015-10-16

**Authors:** Jinbo Xiong, Heping Chen, Changju Hu, Xiansen Ye, Dingjiang Kong, Demin Zhang

**Affiliations:** 1 School of Marine Sciences, Ningbo University, Ningbo, 315211, China; 2 Collaborative Innovation Center for Zhejiang Marine High-efficiency and Healthy Aquaculture, Ningbo, 315211, China; 3 Faculty of Architectural and Civil Engineering and Environment, Ningbo University, Ningbo, 315211, China; 4 Marine Environmental Monitoring Center of Ningbo, State Oceanic Administration (SOA), Ningbo, 315040, China

## Abstract

Understanding the underlying mechanisms that shape the temporal dynamics of a microbial community has important implications for predicting the trajectory of an ecosystem’s response to anthropogenic disturbances. Here, we evaluated the seasonal dynamics of bacterioplankton community composition (BCC) following more than three decades of mariculture disturbance in Xiangshan Bay. Clear seasonal succession and site (fish farm and control site) separation of the BCC were observed, which were primarily shaped by temperature, dissolved oxygen and sampling time. However, the sensitive bacterial families consistently changed in relative abundance in response to mariculture disturbance, regardless of the season. Temporal changes in the BCC followed the time-decay for similarity relationship at both sites. Notably, mariculture disturbance significantly (*P* < 0.001) flattened the temporal turnover but intensified bacterial species-to-species interactions. The decrease in bacterial temporal turnover under long-term mariculture disturbance was coupled with a consistent increase in the percentage of deterministic processes that constrained bacterial assembly based on a null model analysis. The results demonstrate that the BCC is sensitive to mariculture disturbance; however, a bacterioplankton community could adapt to a long-term disturbance via attenuating temporal turnover and intensifying species-species interactions. These findings expand our current understanding of microbial assembly in response to long-term anthropogenic disturbances.

Coastal aquaculture is increasing globally, which has relieved pressure on food supplies and contributed economic benefits^1^. However, intensive mariculture also generates potential damage to coastal environments^2^,^3^. For example, the accumulation of feces and uneaten fish food has resulted in coastal eutrophication and harmful algal blooms^4^,^5^. Recently, the emergence of disease in aquaculture has been determined as being caused by complex interactions among the host, environmental variables, and the surrounding microbiota^6^,^7^. Therefore, to formulate prevention measures, it is important to understand how a microbial community responds to aquaculture-induced disturbance^8^.

The effects of coastal aquaculture on nutrient discharge and phytoplankton composition have been extensively documented^3^,^9^ and generally show adverse effects on a coastal ecosystem, such as increased frequencies of red tide^10^ and occurrences of toxic dinoflagellate^3^. However, taxonomic sorting of phytoplankton requires highly trained specialists and is costly, thus restricting its application in large-scale biomonitoring efforts. By contrast, microbial communities are sensitive to environmental changes, which can be estimated easily and rapidly with high throughput sequencing techniques^11^,^12^,^13^. In particular, there is ample evidence that microbial assemblages are more sensitive than phytoplankton to disturbances^2^,^4^. For example, it has been shown that the composition of bacterial communities in a fish farm is distinct from that of surrounding locations, compared with only weak changes in phytoplankton assemblages^2^,^14^. Indeed, our recent studies have shown that bacterial indicator phylotypes are predictive of shrimp health status^6^,^15^. In addition, the abundance and virulence of pathogenic bacteria can be largely promoted by sudden changes in aquaculture conditions, such as nutrient enrichment and host animal density^16^. These findings suggest the plausibility of using sensitive bacterial assemblages to evaluate the ecological effects resulting from aquaculture disturbance. Indeed, some efforts have been made to investigate the dynamics of a microbial community associated with aquaculture processes. For example, it has been reported that the temporal variations in a bacterioplankton community are predicable to a certain extent during a period of shrimp culture^4^. However, this prediction can be dramatically disrupted by environmental stress^7^,^15^. In addition, currently available information on microbial temporal dynamics is collected from a snapshot or over several weeks in an aquaculture ecosystem^4^,^6^,^7^, which might obscure the real response of microbial communities to disturbance over long-term scales^17^. For these reasons, it is uncertain whether bacterial communities consistently respond to aquaculture disturbance over seasons.

Previous efforts have elegantly shown that rhythmic biogeochemical variables contribute repeatedly seasonal successions in a bacterioplankton community in a pristine salt lake^18^ and in pelagic ecosystems^12^,^19^ over years. This pattern is expected because nutrient availability can drive niche structure through resource partitioning^4^,^13^,^20^. In contrast, we know much less about how biotic interactions (e.g., mutualism and competition) shape microbial communities, although an exploration of species-to-species interactions is desired for a more integrated understanding of community ecology^18^,^21^,^22^. Recently, increasing evidence has shown that microbial assembly is jointly shaped by stochastic and deterministic processes^23^,^24^,^25^. However, active debate is ongoing on the relative contribution of deterministic forces to microbial variations; it is uncertain whether deterministic forces are trivial^24^, or dominant^25^ factors in relative to stochastic factors. As a result, we know little on how, and to what extent, disturbances, i.e., coastal aquaculture, affect temporal dynamics in a microbial assembly, even though this information is central to predicting the trajectory of microbial responses to anthropogenic disturbances^4^,^17^,^21^,^23^. To acquire this knowledge, we collected seawater samples seasonally from a coastal fish farm and its adjacent control site (1) to explore the temporal pattern of a bacterioplankton community and the surrounding water chemistry and to ascertain their relationship; (2) to evaluate to what extent the bacterial temporal turnover, species-species interactions, and relative importance of deterministic processes are altered by mariculture disturbance; and (3) to screen sensitive bacterial assemblages for characterizing such disturbance.

## Results

### Biogeochemical characteristics of water samples

Water temperature monotonically decreased along our sampling seasons (Fig. S1), which was in concert with the temperature trend over the three seasons. The levels of nutrients, such as dissolved organic carbon (DOC), chemical oxygen demand (COD), dissolved inorganic nitrogen (DIN) and PO_4_^3−^, were generally higher at the fish farm site than at the control site (Fig. S1), indicating the presence of a nutrient-enriched environment at the fish farm. In addition, for a given water variable, the temporal change was similar between the two sites; for example, the level of COD peaked in autumn, whereas total phosphate (TP) consistently increased over the seasons at both sites (Fig. S1).

### Effects of mariculture disturbance and sampling time on the BCC

The sequencing efforts yielded four samples with low-quality reads, which were from W148C and W148F; thus, these four samples were removed from further analysis. In total, 239,335 reads remained after being filtered for quality across the residual 44 samples, with 3,873–7,177 bacterial sequences per sample (mean = 5,439 ± 898). The dominant phyla/classes were *Alphaproteobacteria* (mean relative abundance, 23.8%), *Bacteroidetes* (16.0%), *Gammaproteobacteria* (14.9%), *Cyanobacteria* (14.2%), *Actinobacteria* (11.0%) and *Betaproteobacteria* (9.0%), which cumulatively accounted for 88.9% of the bacterial sequences in all samples. In general, the relative abundances of *Cyanobacteria* and *Actinobacteria* decreased, whereas *Bacteroidetes, Gammaproteobacteria* and *Betaproteobacteria* increased over the three seasons (Fig. S2). Notably, the samples were clustered according to the sampling time and habitats (fish farm and control site, with the exception of day 65) even at this coarse level of classification (Fig. S2).

**Figure 1:**
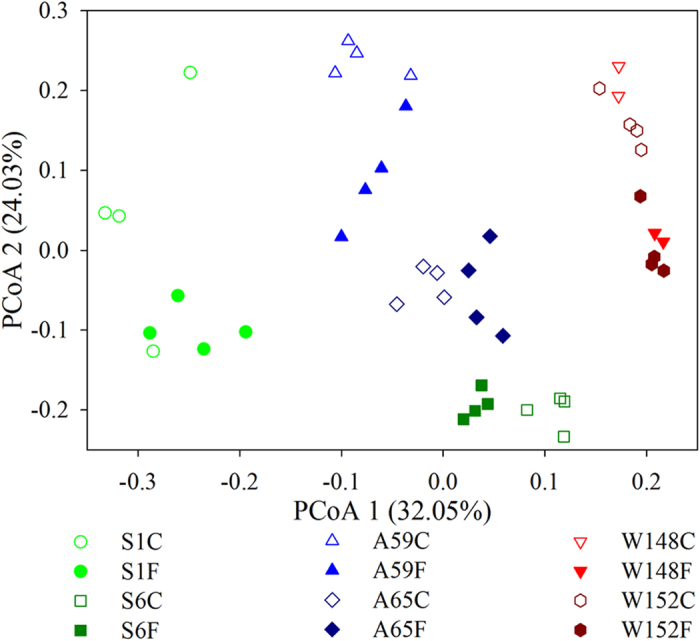
Principal coordinates analysis (PCoA) of the bacterial communities derived from the weighted UniFrac distance matrix. Samples were coded by sampling seasons and habitats. S: summer; A: autumn; W: winter; C: control; F: fish farm. The numbers represent interval days after the first sampling.

The PCoA biplots revealed that the major separation of the bacterioplankton community composition (BCC) was based on the sampling time, though a clear differentiation between mariculture and control site was also evident (Fig. 1). This pattern was further confirmed by PERMANOVA, which showed that the sampling time constrained 19.3% (*P* < 0.001) variation of the BCC, whereas the habitats (fish farm or control site) contributed 4.3% (*P* = 0.008), and no interaction (*P* = 0.403) was detected (Table 1), suggesting that sampling time and mariculture disturbance separately affect the BCC. In addition, the dissimilarity test showed that the bacterial communities were distinct (*P* < 0.05) between the fish farm and control site at each sampling day (with an exception on day 148, Table S1). In contrast, there was no organized pattern of bacterial α-diversity and evenness over the three seasons or between the two sites (Fig. S3). Notably, the relative abundances of potential bacterial predators (*Bdellovibrionales* and *Myxococcales* in our pyrosequencing data)^22^,^26^ were positively associated with the bacterial α-diversity indices (Table S2) and were significantly correlated (Mantel test, *P* = 0.001 for *Bdellovibrionales*, and *P* = 0.013 for *Myxococcales*) with the variations of the BCC.

### Identification of key bacterial families for characterizing mariculture disturbance over seasons

**Figure 2:**
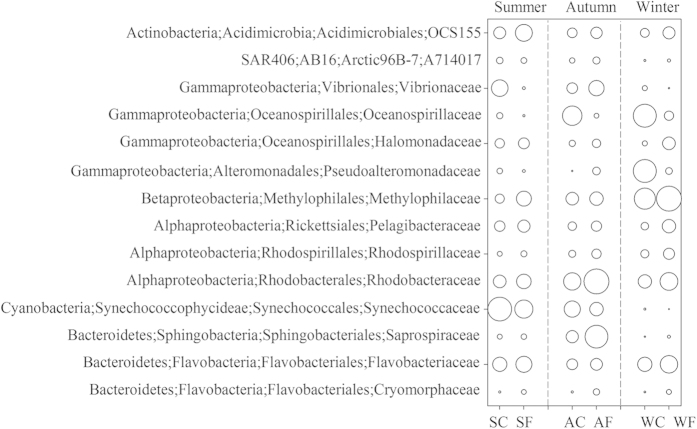
The occurrences of the fourteen dominant bacterial families in the fish farm and control site over the three seasons. The diameters of the circles are proportional to the mean relative abundances (square root transformed) of each family at a given site over the three seasons. S: summer, A: autumn, W: winter, C: control, F: fish farm.

Given that the BCC varied by season, we next asked whether taxa responded to mariculture disturbance in a similar pattern over seasons. Bacterial assemblages at the family level generate the highest ecological cohesion to indicate health status in shrimp aquaculture^15^. Thus, we compared the patterns of change in bacterial assemblages at this taxonomic level. Generally, the patterns of change in the fourteen dominant families (mean relative abundance >1%) were similar (with the exception of *Vibrionaceae* and *Pseudoalteromonadaceae*) between the fish farm and control site over the three seasons, although the seasonal variation was high (Fig. 2). That is, for a given bacterial family, relative abundance consistently increased or decreased at the fish farm site compared with the control site over the seasons. For example, the relative abundances of *Flavobacteriaceae* and *Rhodospirillaceae* were elevated at the fish farm site compared to the control site; in contrast, *Synechococcaceae* and *Oceanospirillaceae* showed an opposite pattern (Fig. 2), indicating that the consistent change patterns of these key bacterial families are tightly associated with mariculture disturbance. Notably, the abundances of these families were significantly correlated with the levels of geochemical variables and biotic factors, i.e., Chl *a* (Table S3).

### Time decay for similarity relationship

**Figure 3:**
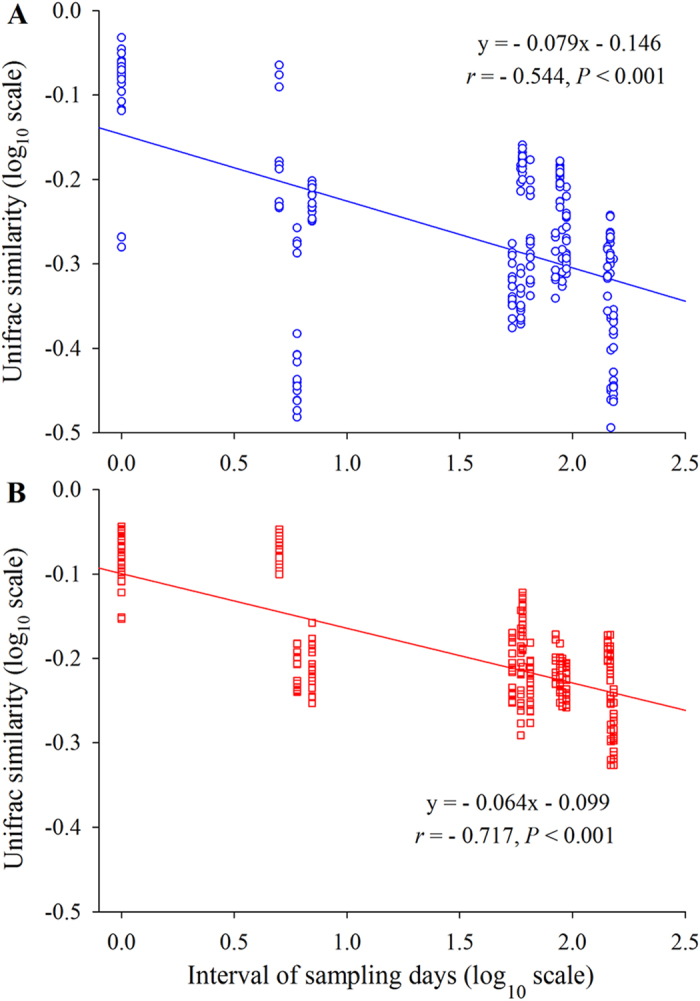
Time-decay for similarity relationship for the bacterial communities at control (A) and fish farm sites (B). The turnover rate, *w* (the regression slope), is estimated using a linear regression (log-log space approach) fit between the pairwise average similarity values and intervals of sampling time.

To evaluate the effects of mariculture disturbance on the temporal turnover of the BCC, we estimated the time-similarity relationship for the fish farm and the control site separately. Significant time decay for similarity of the BCC was detected at both sites (*P* < 0.001, in both cases), with turnover rates of 0.079 at the control site and 0.064 at the fish farm (Fig. 3). Both turnover rates significantly deviated from zero according to a permutation test (*P* < 0.001). Notably, the rate of fish farm samples was significantly shallower (*P* < 0.001) than the samples of the control site. In addition, the similarities of the BCC within the fish farm were significantly higher (0.255 ± 0.102 vs. 0.176 ± 0.112, *P* < 0.001) than within the control site, indicating that there was less variation in the BCC under mariculture disturbance.

### Linkage between bacterial community structure and biogeochemical properties

Significant correlations were detected between the BCC and all environmental variables (*P* < 0.01, Table 2). Of all the water geochemical variables, temperature and DO were the predominant factors shaping the variations of the BCC. In addition, sampling time was found to be significantly associated with the BCC, which was in concert with the time decay for similarity relationship (Fig. 3). Notably, a few factors, such as TN, DIN and Chl *a*, did not improve the regression model in the conditional test; thus, the effects of these factors on the temporal dynamics of the BCC were overridden by other more important variables, i.e., temperature and DO (Table 2).

### Stochasticity versus determinacy under mariculture disturbance

**Figure 4:**
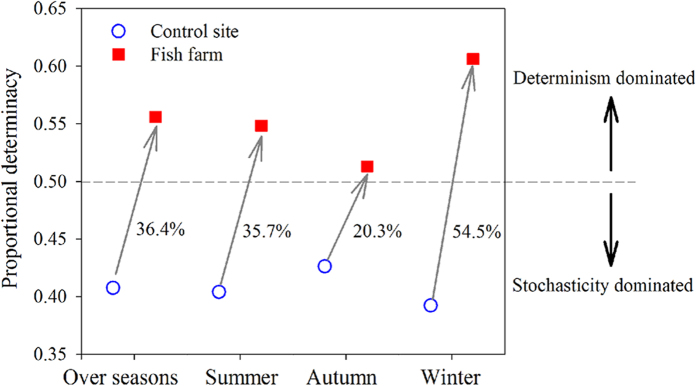
Changes in the percentage of determinacy at the control site (blue open circles) and fish farm (red solid squares) the BCC over seasons. The dashed line denotes equal roles for both. Below the line, stochasticity is dominant, and above it, determinacy is dominant. The numbers associated with each line indicate a percentage increase in determinacy.

Using a null model analysis, we quantitatively estimated changes in the relative importance of stochastic and deterministic processes in driving the temporal dynamics of the BCC (Fig. 4). The percentage of the difference between the observed similarity for each pairwise comparison and the null expected similarity divided by the observed similarity was calculated. The results showed that bacterial assembly processes were constrained by both stochastic and deterministic processes, of which determinacy accounted for 39.2–42.6% and stochasticity accounted for 51.3–60.6%. Notably, the deterministic selection processes of the fish farm were substantially increased in shaping the BCC in relation to those of the control site in each season, increasing 36.4% for all the seasons, 35.7% in summer, 20.3% in autumn, and 54.5% in winter (Fig. 4).

### Networks reveal hubs of network interaction

**Figure 5:**
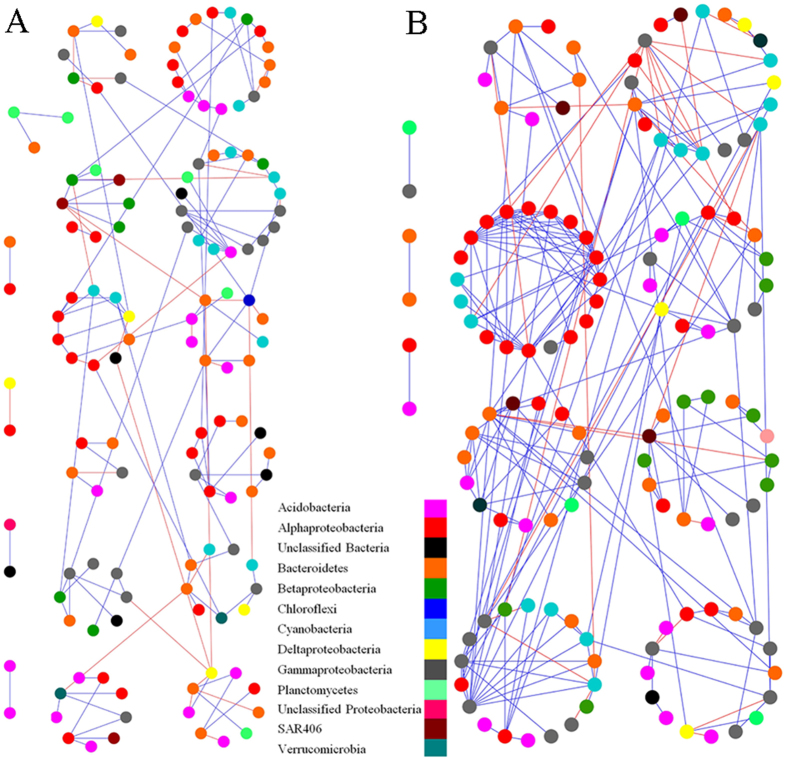
The network graph for control site (A) and fish farm (B) bacterial communities with sub-module structure by the fast greedy modularity optimization method^21^. Each node represents a bacterial OTU. Colors of the nodes indicate OTUs affiliated to different major phyla or classes for *Proteobacteria*. A blue edge indicates a positive interaction, whereas a red edge indicates a negative interaction between two individual nodes.

As previously proposed, we focused on the core OTUs that were detected in more than 50% of the samples in each site^21^, resulting in 189 and 227 OTUs for control and fish farm datasets, respectively. The networks plot revealed that the bacterial species-to-species interactions were more complex and better connected at the fish farm than that at the control site, although a higher module number was detected at the control site (Fig. 5). In addition, there was a higher percentage of positive associations between species at the fish farm than at the control site (87.9% vs. 80.5%). This pattern was further verified by the key network topology parameters, including higher links, average degree and clustering coefficient, and the lower average path (Table 3). This pattern reveals a greater number of interacting species, more complexity, a higher extent of module structure, and closer nodes in the network^21^ of the fish farm bacterial community than in the control site.

## Discussion

The bacterioplankton communities dramatically changed across the three seasons and between sites (Fig. 1), and such variations are closely associated with temperature and geochemical variables (Table 2), matching the notion that temperature and nutrient levels cause pronounced changes in the BCC in coastal waters^4^,^12^,^19^. However, the importance of geochemical variables in driving bacterial communities does not completely rule out the effects of other factors in shaping the variation in the BCC. Indeed, we found that sampling time was a dominant factor in shaping the variation (10.47%) of the BCC, greater than any other geochemical variables with the exception of temperature (Table 2). This pattern is also evident at a coarse taxonomic level (phylum) (Fig. S2). In contrast, no trends were observed for the temporal dynamics of α-diversity, although studies have shown periodic patterns in bacterial diversity over years^12^,^18^. Strikingly, changes in bacterial α-diversity were positively associated with the relative abundances of the potential predators (Table S2). It appears that the potential bacterial predators apparently have no selective predation. Support for this assertion comes from the finding that absence of a given bacterial assemblage is consistent over our study period (Fig. S2). Thus, we posit that the dynamics of the populations may follow a “kill the winner” pattern, as has been observed with phage predation^27^.

*Alphaproteobacteria* species dominated the bacterial communities over our monitored seasons at both sites (Fig. S2). The dominance of this class has been extensively detected in coastal ecosystems^11^,^12^,^19^. *Rhodobacteraceae* (affiliated with *Alphaproteobacteria*) was a dominant bacterial family in our monitored coastal ecosystem, in areas that had recently undergone eutrophication^3^. This pattern is concordant with its ability to better adapt to nutrient-enriched conditions^28^. Unexpectedly, the relative abundance of *Pelagibacteraceae* increased in the fish farm site (Fig. 2), whereas members affiliated with this family appear to be abundant in oligotrophic zones^29^. Notably, the relative abundances of the three dominant families *Saprospiraceae, Flavobacteriaceae* and *Cryomophaceae*, (all affiliated with *Bacteroidetes*), were consistently enriched at the fish farm site compared to the control site over the three seasons (Fig. 2). This pattern is expected, as high similarities in the core genomes of many *Bacteroidetes* species indicate that they have adapted the same life strategy^30^, e.g., capable of particulate organic matter mineralization^20^. In contrast, the change patterns of the bacterial families affiliated with *Gammaproteobacteria* were divergent (Fig. 2), which is in concert with the physiologically diverse features of this group, as previously reported^11^,^31^. The picocyanobacteria of *Synechococcaceae* are typically abundant in oligotrophic oceans, and have evolved to be oligotrophic specialists^19^. Our results corroborate this notion, demonstrating a consistent trend toward a decrease in the relative abundance of *Synechococcaceae* at the fish farm site. Similarly, this pattern has been observed in other near-shore mariculture sites^28^. The members of *Pseudoalteromonadaceae* and *Vibrionaceae* are well-known mariculture opportunistic pathogens^16^, and their occurrences are closely associated with nutrient level. Therefore, *Vibrionaceae* would be expected to be enriched at the mariculture sites. Instead, the relative abundances of both families were only enriched at the mariculture sites in autumn (Fig. 2). However, this pattern corroborates the report that there was a *Vibrio*-induced outbreak of fish disease on Sept. 20, evidenced by a bloom of *Vibrio harveyi* species (Tang *et al.* unpublished data). In addition, the relative abundances of these assemblages are closely associated with water geochemical parameters (Table S3). Collectively, temporal dynamics in the relative abundance for a given dominant bacterial families is consistent over seasons under mariculture disturbance. In particular, the patterns of change are in accordance with the known biological and ecological strategies of these groups and are significantly correlated with biogeochemical variables. Thus, these sensitive assemblages might be served as potential ecological indicators for evaluating mariculture disturbance if the pattern could be confirmed extensively and systematically over spatio-temporal scales in future studies.

Bacterioplankton communities have long been recognized for their high temporal dynamics^4^,^12^,^18^. However, scant evidence exists for evaluating how disturbance affects the temporal turnover and underlying mechanisms, despite the importance of this information in predicting broad-scale environmental changes in the future^17^,^23^. Surprisingly, we found that the temporal turnover rate was significantly attenuated by mariculture disturbance (Fig. 3). This finding is in contrast with a recent meta-analysis, which proposed a lower temporal turnover rate under more physico-chemically stable conditions^17^. It should be noted that our study site has been reared for decades^3^; thus, the type of disturbance would be characterized as “press” (i.e., long-term) rather than as “pulse” (i.e., short-term)^23^,^28^. The pressure disturbance supports the temporal patterns observed here, as shown by largely environmentally constrained variations (Table 2). One possible explanation for this pattern is that disturbance enhances niche selection, which increases microbial mortality and reduces the stochastic processes of diversification^32^,^33^. Consistently, we observed that deterministic processes and stochastic processes jointly constrained the temporal dynamics of the BCC (Fig). However, there was a consistently higher percentage of deterministic drivers in fish farm waters: the mean of 36.4% provided further evidence of the intensification of species sorting under mariculture disturbance. Indeed, a few studies have noted that disturbance alters the relative contribution of stochastic and deterministic processes, such as with wildfire disturbance^34^ and oil contamination^35^. It appears that deterministic processes are dominant under long-term disturbance, such as the pattern observed here and in decades of oil-contaminated soils^35^. Conversely, significantly more stochasticity is detected in soils 4 weeks after wildfire^34^ and in groundwater 4 days after adding emulsified vegetable oil^32^. This pattern is consistent with the idea that the bacterial community is simultaneously structured by stochastic and deterministic factors, although the relative importance of each could vary over time under disturbance^32^,^34^,^36^. Alternatively, local dispersal is intensified by disturbance, such as anthropogenic activities and fish swimming, resulting in a homogeneity of the species pool at the fish farm site. In other word, new species are found more slowly with the increasing temporal extent^17^. Support for this assertion comes from the finding that the deviations of the bacterial community are lower in the mariculture than in the control site (Fig. 3). One might argue that community resistance and resilience could also result in a low turnover rate^37^. However, this might be not the case in this study as the bacterioplankton communities are temporally variable and are distinct between the two sites (Fig. 1). Therefore, the bacterial communities likely have shifted to an alternative stable status in response to disturbance^23^. Nevertheless, once in an alternative equilibrium, it is difficult to return to the previous community composition or function^23^,^38^. Regardless of the cause, future studies are needed to evaluate the ecological consequences of the reduced turnover in bacterial succession.

The interactions among different microbial populations in a community play critical roles in determining an ecosystem’s stability and function^21^,^23^. Strikingly, the bacterial species-to-species interactions were intensified by mariculture disturbance, rather than disrupted (Table 3 and Fig. 5). Less favorable conditions (frequent changes in physicochemical conditions and heavy nutrients in the fish farm site) can create a narrower range of niches^17^, leading to the recruitment or the retention of more ecologically related species^33^,^37^. Consistently, the communities were more phylogenetically convergent at the fish farm relative to the control site; that is, lower dispersion among communities (Fig. 3), and increased network complexity (Fig. 5). As such, it enables a greater stability of the bacterial assemblages^23^,^38^. Similarly, increased phylogenetic clustering among bacterial communities undergoing experimentally induced stresses^23^,^39^,^40^ and convergent phylogenetic relatedness among marine microbial eukaryotic assemblages under increased river discharge^23^,^33^ have been reported. Likewise, the bacterial network interactions are reported to be enhanced by elevated CO_2_ disturbance^21^. Therefore, we infer that intensification of the microbial network interactions might be an alternative mechanism in response to long-term disturbance.

In summary, long-term mariculture disturbance significantly attenuates the temporal turnover of the BCC but intensifies the species-to-species interactions, that is, the enhanced ecological interactions among bacterial assemblages to adapt to disturbance. Consistently, an increase in the deterministic processes shapes the temporal dynamics under mariculture disturbance. Thus, the bacterial community could shift to an alternative stable status in responses to long-term disturbance. In addition, we identified 14 bacterial families that consistently responded to mariculture disturbance, even though the BCC was highly variable over the three seasons. Our study provides insight into the underlying mechanisms that shape the temporal dynamics of the BCC, which is important for predicting the ecosystem responses to anthropogenic disturbance. However, further studies are needed to verify this pattern on a time scale of years to decades.

## Methods

### Study site and sample collection

We collected surface seawater samples (at a depth of 50 cm) in fish-net cages (29° 32′20” N, 121° 45′10” E, with suffix “F”) and an adjacent control site (29° 36′4” N, 121° 46′10” E, 7 km apart from the fish-net cages, with suffix “C”) from a coastal fish farm in Xiangshan Bay (Fig. S4). This bay is a subtropical, eutrophic, semi-enclosed bay connected to the East China Sea, where has been a typical aquaculture zone for three decades^3^. The duration of 90% water exchange is approximately 70 days. Eight cruises were conducted during the spring (April 6 and April 10; due to sample storage problem, these samples were not included in the subsequent analysis), summer (July 13 and July 18, with suffix “S”), autumn (Sept. 10 and Sept. 16, with suffix “A”) and winter (Dec. 07 and Dec. 12, with suffix “W”) in 2012. This experimental design enabled us to explore the temporal dynamics of a bacterioplankton community over days (short-term scale) and seasons (long-term scale). Each four water samples were respectively taken from 4 fish net-cages, and within an area of 30 m × 30 m at the control site. The samples were stored in an icebox and immediately transported to the laboratory within 4 h. In total, we obtained 48 water samples (two sites × four replicates × six time points). We defined the first sampling in the analysis (on July 13) as day 1. Each sample was labeled according to its sampling season, location and interval after the first sampling (i.e., “S1C” means the sample was collected from control site on day 1 in the summer).

Water temperature was recorded *in situ*. Dissolved oxygen (DO) was measured by Winkler titrations^38^. The levels of DOC, total nitrogen (TN), TP, NO_3_^−^, NO_2_^−^, PO_4_^3+^ and COD were analyzed following standard methods^41^. The DIN concentration was calculated as the sum of NH_4_^+^, NO_3_^−^ and NO_2_^−^. The Chl *a* was measured following previously described methods^11^.

### DNA extraction, bacterial 16S rRNA gene amplification and pyrosequencing

On the sampling days, approximately 1 L of water was prefiltered through nylon mesh (100-μm pore size) for use in DNA extraction. The samples were subsequently filtered onto a 0.22-μm membrane (Millipore, Boston, MA, USA) to collect microbial biomass. Genomic DNA was extracted using a MOBIO Power Water DNA isolation kit (MP Biomedicals, Irvine, CA, USA) according to the manufacturer’s protocols. DNA extracts were quantified with a NanoDrop ND-1000 spectrophotometer (NanoDrop Technologies, Wilmington, USA) and were stored at −80 °C until amplification.

An aliquot (50 ng) of DNA from each sample was used as a template for bacterial 16S rRNA gene amplification. The V4–V5 region was amplified using a region-specific primer set: primer F515: GTGCCAGCMGCCGCGG, which contained the Roche 454 “A” pyrosequencing adapter and a unique 11-base barcode sequence, and primer R907: CCGTCAATTCMTTTRAGTTT, which contained the Roche 454 “B” sequencing adapter at the 5′ terminus of the primer^42^. To reduce the effect of random PCR amplification, all samples were amplified in triplicate with the following reaction conditions: 30 cycles of denaturation at 94 °C for 30 s, annealing at 55 °C for 30 s, and extension at 72 °C for 30 s, with a final extension at 72 °C for 10 min^11^. PCR products for each sample were pooled and purified using a QIAquick PCR purification kit (Qiagen, GmbH, Hilden, Germany) and then quantified with a PicoGreen kit (Invitrogen, Carlsbad, CA, USA). Equimolar amounts of PCR products for each sample were combined in a single tube for analysis on a Roche FLX+ 454 pyrosequencer (Roche Diagnostics Corporation, Branford, CT, USA), producing reads from the forward direction (F515 with barcode).

### Processing of pyrosequencing data

Sequencing reads were processed using the Quantitative Insights Into Microbial Ecology (QIIME v1.7.0) pipeline^43^. The sequences were quality filtered on the basis of quality score, sequence length, and primer mismatch thresholds. They were then checked for chimeras and assigned to samples based on an 11-base barcode for each sample. In brief, the homopolymer runs exceeding 6 bp were removed, and chimeras were detected using USARCH^44^. The bacterial phylotypes were identified using UCLUST^45^ and binned into operational taxonomic units (OTUs, 97% similarity). The most abundant sequence in a given OTU was selected as the representative sequence and was aligned using PyNAST^46^. The taxonomic identity of each phylotype was determined using the Greengenes database (release 12.10) taxonomy via the RDP classifier^47^. After taxonomies have been assigned, OTUs affiliated with Archaea, Chloroplasts and unclassified (not affiliated with the domain of Bacteria) were removed from the dataset prior to subsequent analysis. To allow comparison of diversity without bias from unequal sequencing effort, we used a randomly selected subset of 3,870 sequences from each sample in the QIIME pipeline to calculate the diversities and dissimilarities among samples. The sequences generated in this study were deposited in the DDBJ (http://www.ddbj.nig.ac.jp/) Sequence Read Archive under the accession number DRA003584.

### Statistical analysis

Principal coordinates analysis (PCoA) and analysis of similarity (ANOSIM) based on weighted UniFrac distance were performed to evaluate the overall differences in the BCC^48^. The time decay for similarity relationship (TDR) was used to assess the temporal turnover rate of the BCC^4^. To test if the turnover of TDR was different from zero, bootstrapping was applied to regress variables that violated the assumption of independence^49^. The rate was tested by a one-sample *t* test between the original rate and a mean of bootstrapped rates by random pairing of the original set. Molecular ecological networks (MENs) analysis was used to evaluate the effects of mariculture disturbance on the bacterial species-to-species interactions, using an open-accessible pipeline (http://ieg2.ou.edu/MENA). This approach is remarkable in that the network is automatically defined and robust to noise^21^. The network was plotted in Cytoscape 3.1.1^50^.

The following analyses were performed using the “*vegan*” and “*ecodist*” packages in R (version 3.0.2, http://www.r-project.org). Permutational multivariate analysis of variance (PERMANOVA) with ADONIS function was conducted to quantitatively evaluate the contribution of habitats (fish farm and control site) and sampling time to the variations of the BCC^51^,^52^. To determine the factors that significantly constrained the BCC, the environmental variables were selected by a forward selection in a distance-based multivariate linear model (DistLM)^53^. The contribution of each environmental variable was evaluated using “marginal tests” to assess the statistical significance and percentage contribution of each variable taken alone, and then “sequential tests” to evaluate the cumulative effect of the environmental variables explaining the variations in the BCC^54^. The null model analysis was used to evaluate quantitatively the role of deterministic selection processes in shaping the BCC^55^, which was calculated as the proportion of the difference between the observed similarity and the similarity expected under the null hypothesis divided by the observed similarity^56^.

## Additional Information

**How to cite this article**: Xiong, J. *et al.* Evidence of bacterioplankton community adaptation in response to long-term mariculture disturbance. *Sci. Rep.* **5**, 15274; doi: 10.1038/srep15274 (2015).

## Supplementary Material

Supplementary Information

## Figures and Tables

**Table 1:** Quantitative effects of sampling time and habitats (fish farm and control site) on the variation in the BCC with *adonis* 1 based on the Bray-Curtis distance.

	**Sampling time**		**Habitats**		**Sampling time: Habitats**	
	**R**^**2**^	***P***	**R**^**2**^	***P***	**R**^**2**^	***P***
Community structure	0.193	**0.001**	0.043	**0.00118**	0.019	0.403

^1^*adonis*: permutational multivariate analysis of variance (PERMANOVA) with the *adonis* function. The R^2^ value is the proportion of the community variances constrained by the factor.

**Table 2:** Results of a distance-based multivariate linear model for the BCC showing the percentage of variation explained by individually environmental variables (ignoring other variables); and forward-selection of variables, where amount explained by each variable added to model is conditional on variables already in the model.

**Variable**	***F***	***P***	**Variation (%)**	**Cumulative (%)**
Variables fitted individually				
Temperature	10.71	0.001	20.31	
Sampling time	10.01	0.001	19.25	
Total phosphorus	9.15	0.001	17.88	
Dissolved oxygen	8.47	0.001	16.78	
Chemical oxygen demand	6.96	0.001	14.22	
PO_4_^3−^	6.43	0.001	13.28	
C/N ratio	6.15	0.001	12.78	
Chlorophyll *a*	6.01	0.001	12.51	
NO_3_^−^	5.95	0.001	12.42	
NH_4_^+^	5.76	0.001	12.05	
DIN	5.10	0.001	10.83	
Total nitrogen	4.40	0.001	9.48	
NO_2_^−^	3.55	0.002	7.80	
Total organic carbon	2.62	0.008	5.87	
Variables fitted sequentially				
Temperature	10.71	0.001	20.31	20.31
Sampling time	6.81	0.001	10.47	30.79
Dissolved oxygen	6.21	0.001	10.06	40.85
C/N ratio	3.87	0.001	5.34	46.19
NO_2_^−^	3.97	0.001	5.09	51.28
Total organic carbon	4.22	0.001	4.99	56.27
PO_4_^3−^	3.61	0.001	3.99	60.26
Total phosphorus	3.41	0.001	3.53	63.79
NO_3_^−^	3.53	0.001	3.40	67.20
NH_4_^+^	3.39	0.001	3.06	70.25
Chemical oxygen demand	2.37	0.001	2.05	72.30

NOTE: Program ended prematurely because not all variables increased the regression model in the conditional tests. DIN: dissolved inorganic nitrogen, the sum of NH_4_^+^, NO_3_^−^ and NO_2_^−^.

**Table 3:** Topological properties of the empirical molecular ecological networks (MENs) of bacterial communities and their associated random MENs.

	**Topological properties**	**Habitats of communities**	
		**Control**	**Fish farm**
Empirical networks	Similarity threshold (st)	0.92	0.9
	Network size (n)	133	134
	Links (n)	150	224
	R^2^ of power law	0.813	0.907
	Average path (GD)	4.637	3.955
	Average Clustering coefficient (avgCC)	0.058	0.132
	Average degree (avgK)	2.256	3.343
	Modularity (M)	0.744	0.642
Random networks	Average path (GD)	4.637 ± 0.562	3.521 ± 0.205
	Average Clustering coefficient (avgCC)	0.011 ± 0.008	0.033 ± 0.011
	Modularity (M)	0.719 ± 0.013	0.527 ± 0.011
