# Supplementary material for: Evidence of bacterioplankton community adaptation in response to long-term mariculture disturbance
Source: Sci Rep. 2015 Oct 16;5:15274. doi: 10.1038/srep15274 (PMC4607939; doi:10.1038/srep15274)

Supplemental Information

Evidences of bacterioplankton community adaption in responses to long-term mariculture disturbance

Jinbo Xiong, Heping Chen, Changju Hu, Xiansen Ye, Dingjiang Kong
& Demin Zhang

A. Supplemental Tables

Table S1 Community dissimilarity test by analysis of similarity (ANOSIM) based on Bray-Curtis distance, pair-wise *P* values were shown

Table S2 Pearson correlations between the relative abundances of potential predators and bacterial **α**-diversity

Table S3 Spearman rank coefficients between the relative abundances of 14 dominant bacterial families and selected biogeochemical factors across the samples. Only the significant coefficients ($P < 0.05$) are listed here

B. Supplemental Figures

Figure S1 Summary of the water biogeochemical variables across the sampling time. Error bar indicates a standard deviation within each site ($N = 4$)

Figure S2 Heat map of the abundant bacterial phyla (relative abundance > 1%, *Proteobactria* were classified into class level) across the samples

Figure S3 The temporal dynamics of bacterial **α**-diversity: Shannon diversity (A) and phylogenetic diversity (B) over the sampling date in both mariculture and control sites

Figure S4 Map of fish farm and control site in Xiangshan Bay showing the sampling locations

Table S1 Dissimilarity test by analysis of similarity (ANOSIM) based on Bray-Curtis distances between bacterial communities, pair-wise *P* values were shown

	S1C	S1F	S6C	S6F	A59C	A59F	A65C	A65F	W148C	W148F	W152C	W152F
S1C												
S1F	**0.0267**											
S6C	0.0245	0.0285										
S6F	0.0080	0.0089	**0.0071**									
A59C	0.0286	0.0301	0.0274	0.0081								
A59F	0.0275	0.0301	0.0268	0.0083	**0.0278**							
A65C	0.0280	0.0251	0.0287	0.0069	0.0305	0.0261						
A65F	0.0302	0.0294	0.0268	0.0087	0.0271	0.0265	**0.0297**					
W148C	0.0659	0.0700	0.0631	0.0459	0.0683	0.0674	0.0670	0.0651				
W148F	0.0650	0.0677	0.0714	0.0500	0.0642	0.0659	0.0655	0.0661	0.3335			
W152C	0.0269	0.0308	0.0306	0.0077	0.0292	0.0279	0.0281	0.0304	0.0646	0.0691		
W152F	0.0284	0.0312	0.0284	0.0074	0.0293	0.0286	0.0267	0.0297	0.0694	0.1326	**0.0295**	

The samples are named as season + sampling time + region. The first sampling is termed as day 1.

S: summer; A: autumn; W: winter; C: control; F: fish farm.

The bold values indicate significant differences ($P < 0.05$) in the bacterial communities between control and fish farm sites at each sampling day.

Table S2 Pearson correlations between the relative abundances of potential predators and bacterial **α**-diversity

Predator	Phylotypes		Phylogenetic diversity		Shannon diversity	
	r	*P*	*r*	*P*	*r*	*P*
Bdellovibrionales	0.364	**0.014**	0.397	**0.007**	0.286	**0.047**
Myxococcales	0.765	< **0.001**	0.813	< **0.001**	0.669	< **0.001**

Bold values indicate significant (*P* < 0.05) correlations.

Table S3 Spearman rank coefficients between the relative abundances of 14 dominant bacterial families and selected biogeochemical factors across the samples. Only the significant coefficients ($P < 0.05$) are listed here

Family level	Temperature		DIN		COD		TOC		N/P ratio		Chl *a*	
	ρ	*P*	*ρ*	*P*	*ρ*	*P*	*ρ*	*P*	*ρ*	*P*	*ρ*	*P*
Actinobacteria;OCS155									0.453	0.002		
SAR406;A714017							0.334	0.027			-0.327	0.030
Rhodobacteraceae	0.349	0.020			0.393	0.008						
Rhodospirillaceae	-0.607	< 0.001	0.675	< 0.001					-0.593	< 0.001	-0.628	< 0.001
Pelagibacteraceae												
Cryomorphaceae	0.762	< 0.001	-0.639	< 0.001	0.489	0.001	0.462	0.002	0.438	0.003	0.412	0.006
Flavobacteriaceae									0.274	0.042		
Saprospiraceae	0.263	0.043			0.660	< 0.001	0.466	0.001	-0.288	0.044		
Methylophilaceae	-0.865	< 0.001	0.680	< 0.001	-0.536	< 0.001	-0.260	0.049	-0.597	< 0.001	-0.620	< 0.001
Synechococcaceae	0.589	< 0.001	-0.432	0.003	0.502	0.001			0.453	0.002	0.638	< 0.001
Pseudoalteromonadaceae	-0.278	0.048	0.359	0.017			0.321	0.034	-0.571	< 0.001	-0.621	< 0.001
Halomonadaceae			0.525	< 0.001								
Oceanospirillaceae	-0.421	0.004	0.401	0.007					-0.711	< 0.001	-0.573	< 0.001
Vibrionaceae					0.258	0.049			-0.518	< 0.001	-0.394	0.008

DIN: dissolved inorganic nitrogen, the sum of NH_4^+, NO_3^- and NO_2,

COD: chemical oxygen demand, TOC: total organic carbon,

Chl *a*: chlorophyll *a*

Figure S1 Summary of the water biogeochemical variables across the sampling time. Error bar indicates a standard deviation within each site (N = 4). DO: dissolved oxygen, COD: chemical oxygen demand, TOC: total organic carbon, TN: total nitrogen, TP: total phosphorus, DIN: dissolved inorganic nitrogen, the sum of NH_4^+, NO_3^- and NO_2, Chl *a*: chlorophyll *a*. For sample names, S: summer, A: autumn, W: winter, the numbers represent duration after the first sampling (with suffix "1").

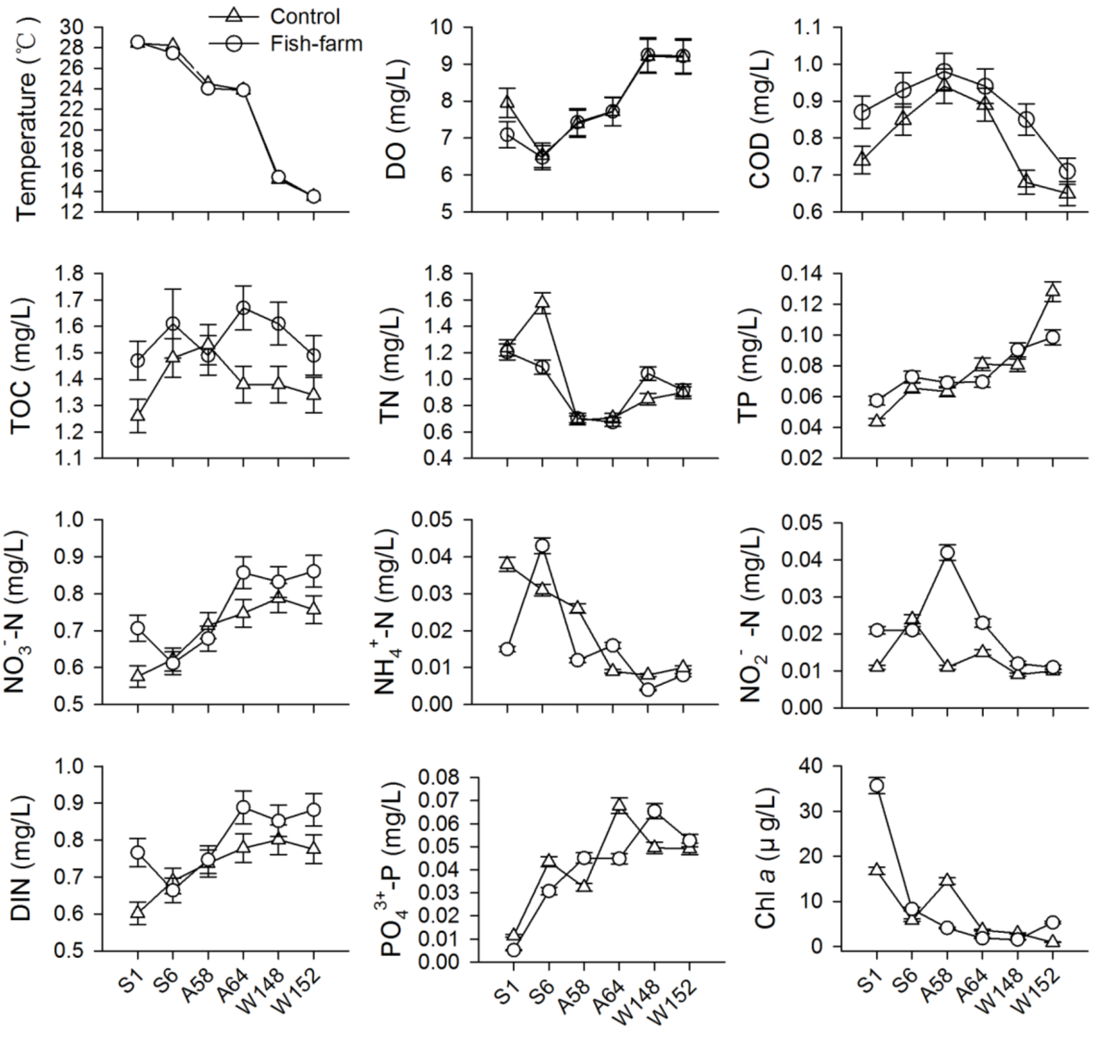

Figure S2 Heat map of the abundant bacterial phyla (relative abundance > 1%, Proteobactria were classified into class levels) across the samples. Temporally collected samples were labeled with the sampling day. Color legend presents bacterial relative abundance. S: summer, A: autumn, W: winter, C: control, F: fish farm. The first sampling day termed as day 1.

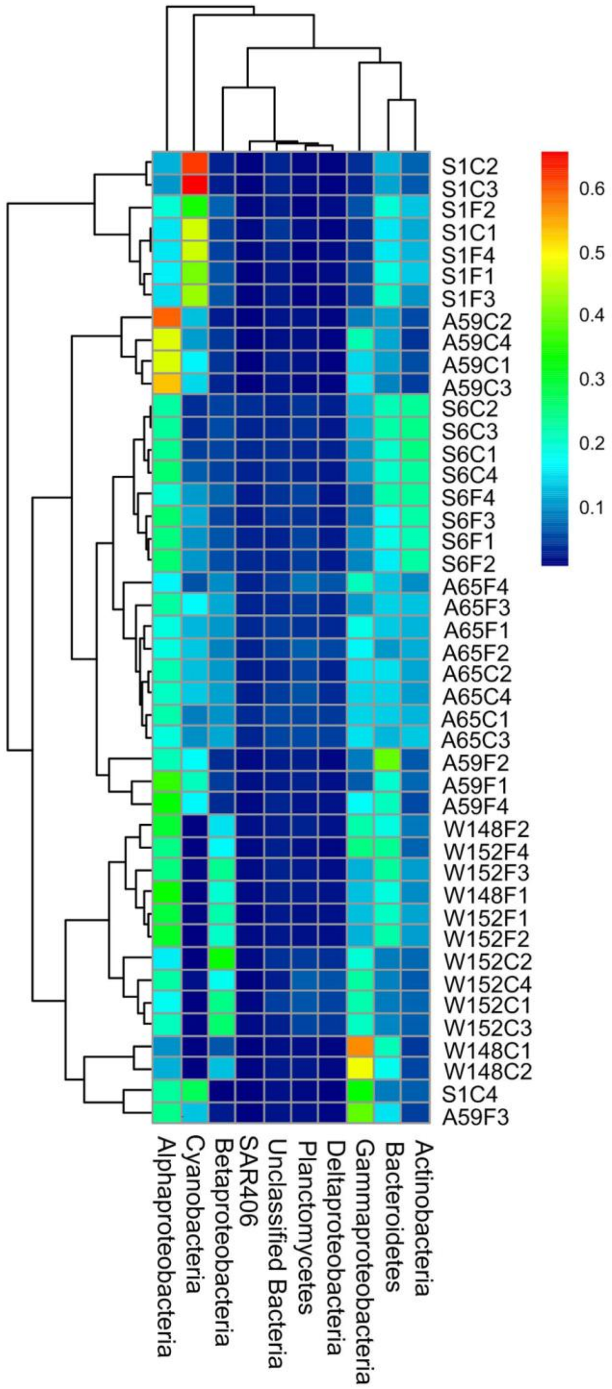

Figure S3 The temporal dynamics of bacterial **α**-diversity: Shannon diversity (A) and phylogenetic diversity (B) over the sampling date in both mariculture and control sites. S: summer, A: autumn, W: winter. The first sampling termed as day 1.

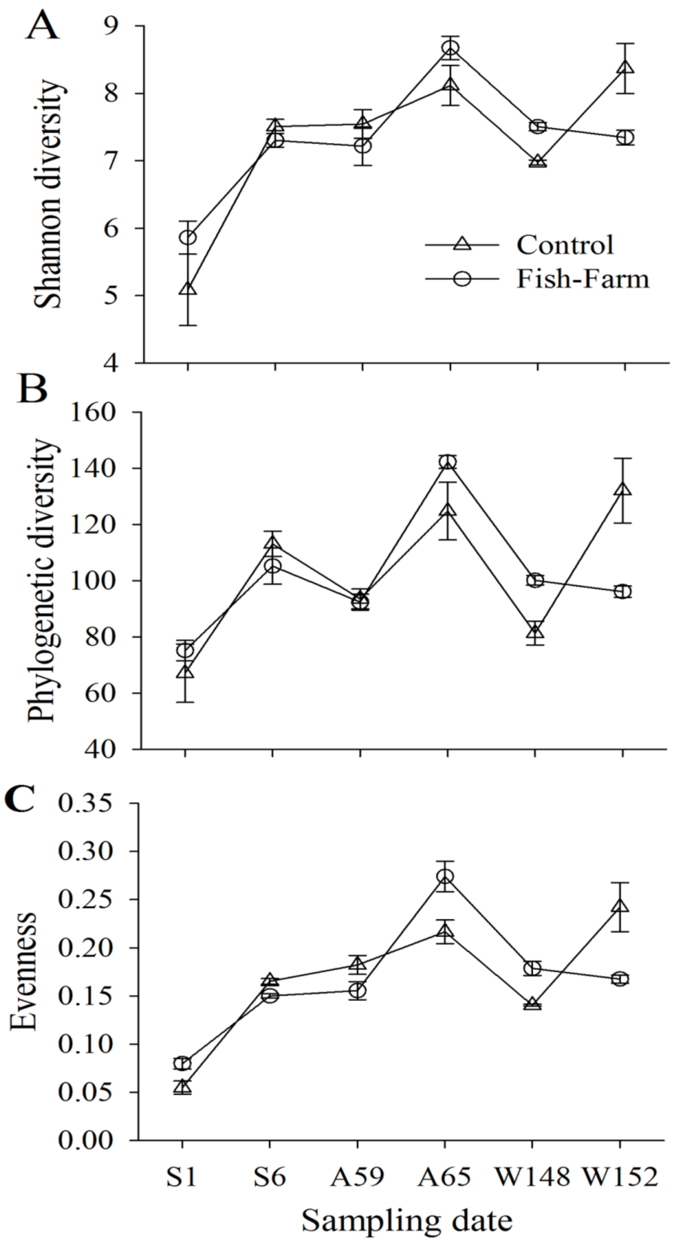

Figure S4 Map of fish farm and control site in Xiangshan Bay showing the sampling locations. The base-map was downloaded from the national geophysical data center (http://www.ngdc.noaa.gov/, open accessible), then we created the map in Surfer 10 software (Golden software, Golden, CO, USA)

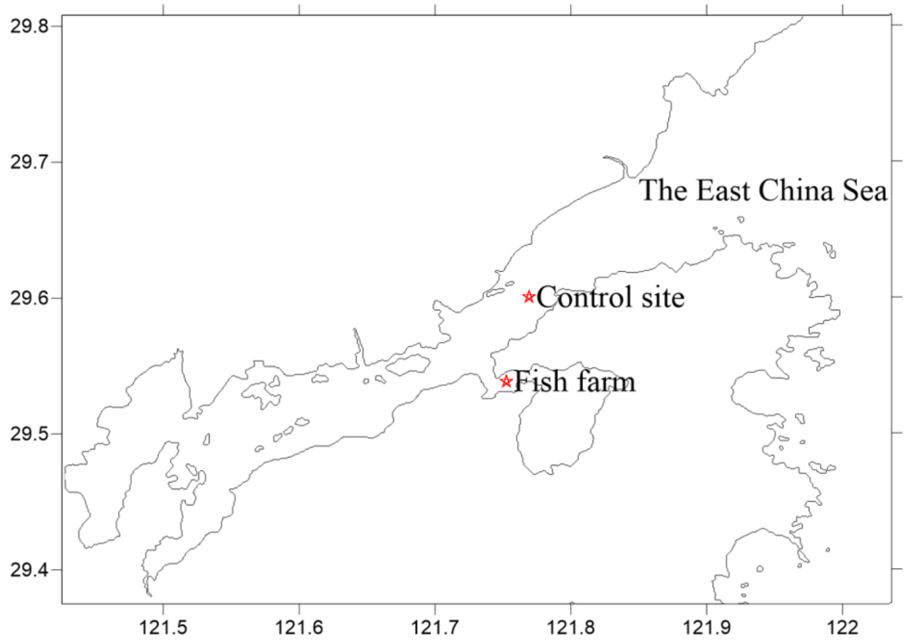

Supplement: Supplementary Information [file srep15274-s1.pdf]
